# The Dual Cell Cycle Kinase Inhibitor JNJ-7706621 Reverses Resistance to CD37-Targeted Radioimmunotherapy in Activated B Cell Like Diffuse Large B Cell Lymphoma Cell Lines

**DOI:** 10.3389/fonc.2019.01301

**Published:** 2019-11-29

**Authors:** Gro Elise Rødland, Katrine Melhus, Roman Generalov, Sania Gilani, Francesco Bertoni, Jostein Dahle, Randi G. Syljuåsen, Sebastian Patzke

**Affiliations:** ^1^Department of Radiation Biology, Institute for Cancer Research, Norwegian Radium Hospital, Oslo University Hospital, Oslo, Norway; ^2^Research and Development, Nordic Nanovector ASA, Oslo, Norway; ^3^Lymphoma and Genomics Research Program, Institute of Oncology Research, Università Della Svizzera Italiana, Lugano, Switzerland

**Keywords:** radioimmunotherapy (RIT), CD37, combination therapy, lymphoma, radiation resistance, aurora kinase, cyclin-dependent kinase, polo-like kinase

## Abstract

The CD37 targeting radioimmunoconjugate ^177^Lu-lilotomab satetraxetan (Betalutin) is currently being evaluated in a clinical phase 2b trial for patients with follicular lymphoma (FL) and in a phase 1 trial for patients with diffuse large B-cell lymphoma (DLBCL). Herein we have investigated the effect of ^177^Lu-lilotomab satetraxetan in seven activated B-cell like (ABC) DLBCL cell lines. Although the radioimmunoconjugate showed anti-tumor activity, primary resistance was observed in a subset of cell lines. Thus, we set out to identify drugs able to overcome the resistance to ^177^Lu-lilotomab satetraxetan in two resistant ABC-DLBCL cell lines. We performed a viability-based screen combining ^177^Lu-lilotomab satetraxetan with the 384-compound Cambridge Cancer Compound Library. Drug combinations were scored using Bliss and Chou-Talalay algorithms. We identified and characterized the dual-specific CDK1/2 and AURA/B kinase inhibitor JNJ-7706621 as compound able to revert the resistance to RIT, alongside topoisomerase and histone deacetylases (HDAC) inhibitors.

## Introduction

Diffuse large B-cell lymphoma (DLBCL) is the most common type of lymphoma, accounting for ~35% of all newly diagnosed cases. Despite its phenotypical relatively homogenous appearance, DLBCL is a heterogeneous diseases (1). So far, the most commonly used sub-classification is based on the cell-of-origin (COO), identifying the germinal center B-cell (GCB)-type and the activated B-cell (ABC)-like DLBCL subtypes on the basis of gene or protein expression pattern reminiscent of normal germinal center or ABCs, respectively. ABC-DLBCL is associated with a worse outcome than GCB-DLBCL when patients are treated with the standard R-CHOP-like treatment (R-CHOP: a combination of the CD20-targeting antibody rituximab and the chemotherapeutics cyclophosphamide, doxorubicin, prednisolone, and vincristine).

Despite the major improvements in our understanding of the biology of DLBCL and the availability of a large number of novel compounds, no new regimen has shown superiority to R-CHOP (2–4). Recent studies have indeed uncovered a high degree of genetic heterogeneity even within the GCB and ABC-DLBCL subtypes (5, 6), thus indicating the need to target more specific subgroups of patients. Furthermore, over-expression of MYC and BCL2 proteins in the absence of chromosomal rearrangements identifies a subgroup of cases, the so-called double expressor lymphomas (DEL), with a particularly poor prognosis. DELs are more frequent in ABC than GCB-DLBCL (1, 7). In standard clinical practice first-line R-CHOP-like treatment of DEL includes etoposide in addition to prednisone, vincristine, cyclophosphamide and doxorubicin (R-ECHOP) [reviewed in (1, 8)]. Patients with refractory, relapsed or treatment resistant DLBCL are, if eligible, treated with intensive chemotherapy [in the case of chemoresistance: rituximab in combination with ifosfamide, carboplatin, and etoposide (R-ICE), or dexamethasone, high-dose cytarabine, and cisplatin (R-DHAP)] followed by autologous (or allogeneic) stem cell transplantation (ASCT). Still, the DEL patient group remain a particularly poor prognostic group with 5-year progression free survival (PFS) of <30% after relapse.

Radioimmunotherapy (RIT) is an alternative and target-specific treatment for lymphomas. RIT is based on the conjugation of a short-ranged and short half-lived radioemitter to a lineage-specific monoclonal antibody (9). ^90^Y-Ibriumomab and ^131^I-tositumomab are examples of CD20 targeted FDA-approved, first-generation RITs for the treatment of relapsed or refractory follicular lymphoma (r/r-FL) and transformed FL [for a review (10)]. These showed promising results for treatment of FL and DLBCL, but, amongst other, logistic challenges resulted in underusage (11–14). ^131^I-tositumomab was withdrawn from the market in 2014. CD37 is a transmembrane protein expressed almost exclusively on cells of the immune system, especially in mature B cells and B cell NHL (15), hence being an important alternative for CD20-targeting therapies, to which treatment-resistance can be developed (16). ^177^Lu-lilotomab satetraxetan (Betalutin®) is a CD37-specific murine monoclonal antibody (clone HH1) that is chelated via a p-SCN-benzyl-DOTA-linker (satetraxetan) to the β-emitting isotope ^177^Lutetium (T_1/2_ = 6.7 days) (17–19). ^177^Lu-lilotomab satetraxetan is currently being investigated as a single-injection mono-therapy for treatment of relapsed or refractory (r/r) FL (NCT01796171, Phase 2B) and r/r-DLBCL (NCT02658968, Phase 1), showing a promising overall response rate in r/r-FL of about 65–70% (20). ^177^Lu-lilotomab satetraxetan may thus have potential in the treatment of high-grade DLBCL, though as in the case of r/r-FL treatment, resistance may occur. To explore and attempt to over-come the potential resistance we chose to investigate the sensitivity to ^177^Lu-lilotomab in cell lines derived from ABC-DLBCL, the DLBCL subtype that has the lower sensitivity to standard regimens. Subsequently, we conducted a combinatorial drug screen for small molecular anti-cancer compounds preventing ^177^Lu-lilotomab satetraxetan treatment resistance in the most resistant cell lines. Herein, we report the identification and pre-clinical characterization of a dual CDK1/2 and AURKA/B kinase inhibitor that was identified in the screen as a candidate compound to overcome ^177^Lu-lilotomab satetraxetan therapy resistance.

## Results

### Resistance of U-2932 and RIVA to CD37-Targeted ^177^Lu-Radioimmunotherapy

**Figure 1:**
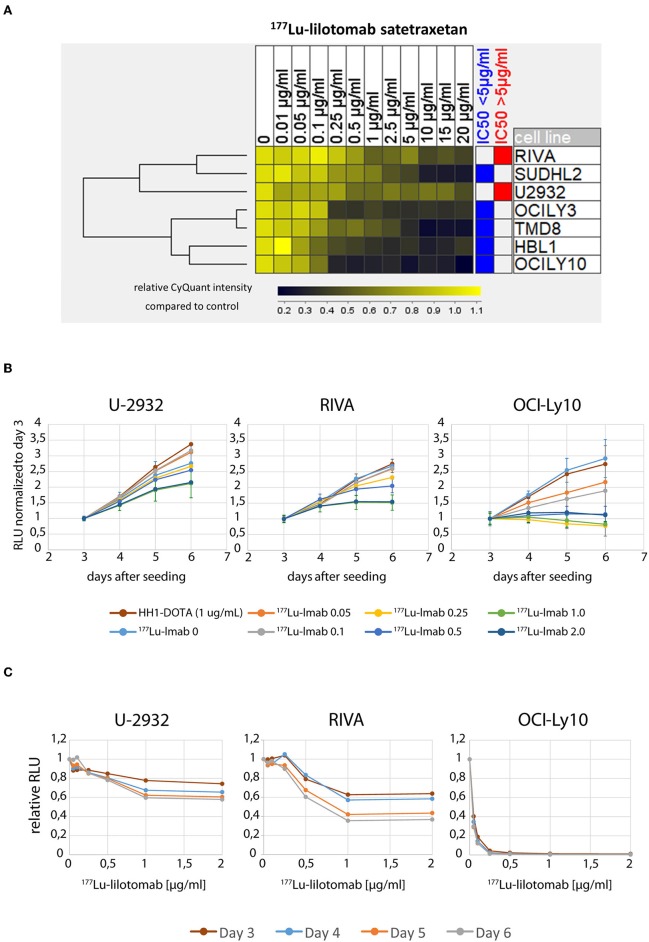
U-2932 and RIVA are resistant to CD37-targeted ^177^Lu-radioimmunotherapy. **(A)** Cells were treated for 18 h with 11 different doses of ^177^Lu-lilotomab satetraxetan ranging from 0.01 to 20 μg/mL (specific activity: 600 MBq/mg), washed and plated in 96-well plates. Mock treated cells were included as control. The total DNA content in each well was assessed using the CyQuant reagent as an equivalent of cell proliferation. **(B,C)** Treated as in **(A)** with doses of ^177^Lu-lilotomab satetraxetan ranging from 0 to 2 μg/mL or cold antibody (HH-1-Dota) and measuring proliferation utilizing MT, RealTime-Glo, adding luminescent assay substrate 72 h after seeding in micro-well titer plates. **(C)** Relative RLU (^177^Lu-lilotomab satetraxetan to control) of data presented in **(B)**. Error bars: Standard deviation (STDEV) (*n* = 5 for U-2932 and RIVA, *n* = 3 OCI-Ly10). Inhibition of cell proliferation on days 5 and 6 were significantly reduced compared to control (*p* < 0.001, 1-way ANOVA) in U-2932 cells at doses ≥ 1 μg/mL, in RIVA at doses ≥ 0.25 μg/mL, and Oci-Ly10 at doses ≥ 0.1 μg/mL.

We initially investigated the sensitivity of seven different ABC-DLBCL cell lines (HBL1, OciLy-3, Oci-Ly10, RIVA [RI-1], SU-DHL-2, TMD-8, U-2932) to treatment with ^177^Lu-lilotomab satetraxetan. Cells were treated for 18 h with 11 different doses of ^177^Lu-lilotomab satetraxetan ranging from concentrations of 0.01–20 μg/ml (specific activity: 600 MBq/mg), washed and plated in 96-well plates. Mock treated cells were included as controls. The total DNA content in each well was assessed using the CyQuant reagent as a readout of cell proliferation. Comparative analysis of relative proliferation capacity compared to untreated control cells identified U-2932 and the RIVA cells as the most resistant cell lines, showing over 40% of signal intensity of untreated control cells even after treatment at 20 μg/ml ^177^Lu-lilotomab satetraxetan (Figure 1A). Conversely, Oci-Ly10 cells showed highest sensitivity to treatment with a 70% decreased proliferation capacity in response to treatment with 0.25 μg/ml ^177^Lu-lilotomab satetraxetan. Notably, *CD37* mRNA and CD37 surface expression were not associated with the resistance to CD37-target RIT (Table 1). We confirmed the differential sensitivity of these three cell lines in a metabolic cell viability assay, utilizing MT RealTimeGlo, that allowed the monitoring of cell proliferation throughout a continuous period of 72 h (Figures 1B,C). Cells were treated as previously and the luminescent assay substrate added 72 h after plating into micro-well titer plates. All cell lines and control treatment groups showed continuous proliferation throughout the observation period. Addition of cold, non-^177^Lu chelated lilotomab (HH1-DOTA) did not markedly inhibit proliferation in either cell line. Oci-Ly10 cells were sensitive to even the lowest tested dose of 0.05 μg/ml ^177^Lu-lilotomab satetraxetan and ceased proliferation at 0.25 μg/ml. Confirming the observed resistance in the CyQuant assay, U-2932 and RIVA retained ~60 and 40%, respectively, of the proliferation capacity of untreated cells at 5 days after treatment with 2 μg/ml ^177^Lu-lilotomab satetraxetan. Again, RIVA cells were more sensitive to ^177^Lu-lilotomab satetraxetan than U-2932 and showed about 60% of the proliferation capacity of control cells at a dose of 0.5 μg/ml, which is half of the dose required in U-2932 cells to reach a similar level of inhibition.

**Table 1:** Characteristics of ABC-DLBCL cell lines.

**Cell line**	**IC50 (CyQUANT) [μg/ml]**	**CD37 mRNA [RPKM]**	**CD37 mRNA (rel. to RIVA)**	**CD37 protein MS [counts]**	**CD37 protein (rel. to RIVA)**	**CD37 surface HH1-DOTA binding rel. to RIVA**	***TP53***	***MYC***	***BCL2***
Oci-Ly10	0.3	10.6	0.9	29.76	0.99	0.32 ± 0.04	Inactive	WT	WT
HBL1	0.6	11.3	1.0	30.83	1.02	n.d.	Inactive	WT	WT
OCI-LY3	1.1	11.5	1.0	31.08	1.03	n.d.	WT	WT	Amp
TMD8	1.1	11	1.0	31.45	1.04	n.d.	WT	WT	WT
SUDHL2	2.5	9.6	0.8	29.34	0.97	n.d.	WT	WT	WT
RIVA	8.6	11.5	1	30.14	1	1	Inactive	T	Amp
U-2932	31.6	9.4	0.8	29.99	1.00	0.55 ± 0.02	Inactive	OE	Amp

*WT, wild-type; T, translocated; OE, overexpressed, Amp, amplified; RPKM, Reads per kilo base per million (21–23)*.

To conclude, U-2932 and RIVA have been shown to be ^177^Lu-lilotomab satetraxetan treatment resistant ABC-DLBCL cell lines. Furthermore, the resistance of these cell lines to CD37-targeted RIT was not due to a reduced expression of and binding to CD37 on the cell surface.

### Combinatorial Drug Screen Identifies Cell Cycle Kinase Inhibitors as Candidate Drugs to Overcome Radioimmunotherapy Resistance

**Figure 2:**
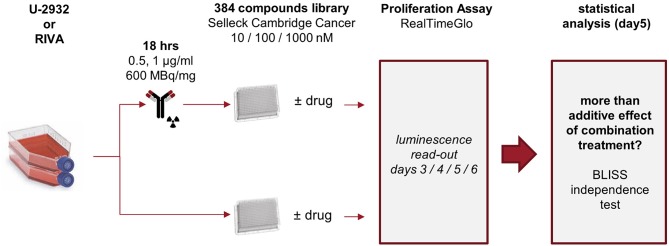
Experimental layout. U-2932 and RIVA cells were treated with 1 or 0.5 μg/mL ^177^Lu-lilotomab satetraxetan (600 MBq/mg) for 18 h, excess ^177^Lu-lilotomab satetraxetan removed, and cells seeded onto 384-well plates pre-printed with the 384-compound Cambridge Cancer compound library sourced from Selleckchem at final concentrations of 10 nM, 100 nM, or 1 μM. Untreated control cells were seeded on parallel plates. Viability measurements, using RealTime-Glo, were carried out between day 3 and 6 after seeding. Inhibitory compounds were considered as candidate hits if they: (1) in combination with ^177^Lu-lilotomab satetraxetan inhibited cell proliferation over two consecutive days to a degree greater than the expected additive effect of the mono-treatments alone (BLISS theorem, see Materials and Methods for details), and (2) drug treatment alone did not reduce viability to <90% of that of untreated control.

Only two out of seven cell lines of the ABC-DLBCL panel did not respond well to CD37-targeted RIT. These two cell lines, U-2932 and RIVA, may represent models of most challenging to treat DLBCL. To understand and overcome the lack of response to ^177^Lu-lilotomab satetraxetan treatment, we set out to find combinatorial drug partners for ^177^Lu-lilotomab satetraxetan capable of reversing CD37-targeted RIT resistance in both cell lines. U-2932 and RIVA cells were treated with 1 and 0.5 μg/ml of ^177^Lu-lilotomab satetraxetan for 18 h, respectively, washed and seeded onto micro-well plates pre-printed with a library of 384 anti-cancer compounds (Cambridge anti-Cancer Compound library; SelleckChem). Three days post-plating RealTimeGlo reagent was added and luminescence read on three consecutive days to assess the relative amount of metabolically active cells. The screen design is schematically presented in Figure 2.

**Figure 3:**
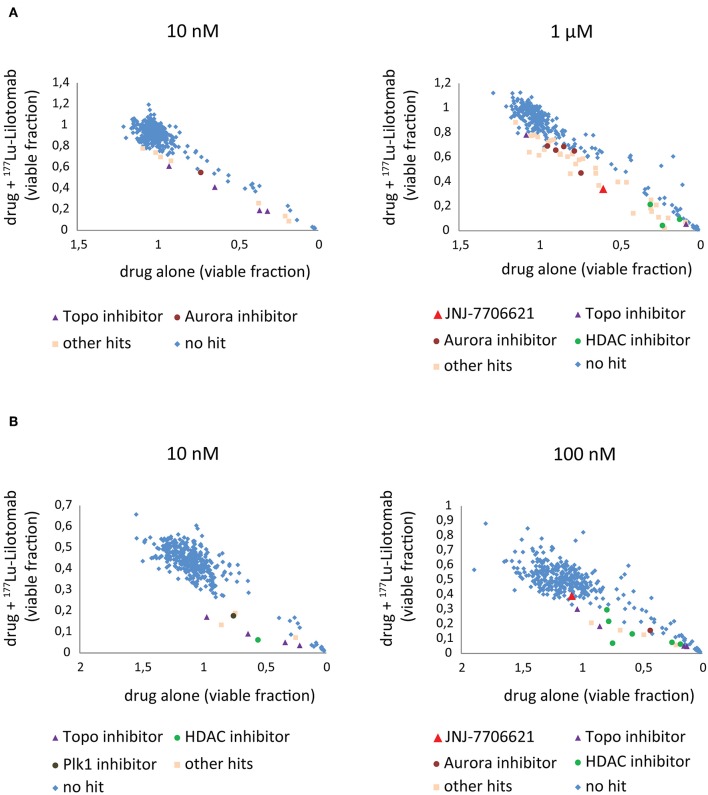
Combination screen for compounds reversing ^177^Lu-lilotomab satetraxetan resistance in DLBCL. Cells were treated as explained in Figure 2. The fraction of viable cells relative to untreated control at day 5 has been plotted for cells treated with drug alone (x-axis) against cells treated with drug combined with ^177^Lu-lilotomab satetraxetan (y-axis) for the two concentrations of drugs tested in each cell line. **(A)** U-2932 and **(B)** RIVA. In blue are drugs excluded as hits by our statistical criteria (See Figure 2). Enriched hits are specified with name of common target and are in separate colors [purple triangles: topoisomerase inhibitors, burgundy circles: aurora kinase inhibitors (including Alisertib) and green circles: histone deacetylase inhibitors]. In red is the pan-CDK1/2 and AuroraA/B inhibitor JNJ-7706621 and in brown the Plk1 inhibitor GSK461364. In light pink are all other hits.

Both cell lines continuously proliferated throughout the observation period, albeit U-2932 cells showed a higher growth rate and stronger resistance to ^177^Lu-lilotomab satetraxetan monotreatment than RIVA cells in the primary screen (Supplementary Figure 1). Three different concentrations of drugs (U-2932: 10/1,000 nM; RIVA: 10/100 nM final concentration) were tested to account for compound-specific differences in potency. Inhibitory compounds were considered as a hit candidate if they: (1) in combination with ^177^Lu-lilotomab satetraxetan inhibited cell proliferation over two consecutive days to a degree greater than the expected additive effect of the mono-treatments alone (Bliss theorem, see Materials and Methods for details), and (2) the inhibitory effect of the compound alone at the tested concentration was <90% relative to the untreated control. These criteria were met by 53 compounds in U-2932 (11 at 10 nM and 42 at 1 μM) and 27 compounds in RIVA cell lines (8 at 10 nM and 19 at 100 nM) (Tables 2, 3). Figures 3A,B summarize the screen results for each cell line and library concentration. Hit candidates are highlighted in dot-plots showing the relative proliferation of cells at day 5 treated with the compound alone vs. its combination with ^177^Lu-lilotomab satetraxetan (datasets are included as excel spreadsheets in Supplementary Material). Topoisomerase inhibitors accounted for 13% of the hits in U-2932 and 23% in RIVA, and histone deacetylase (HDAC) inhibitors for 7% of the hits in U-2932 and 27% in RIVA cells (Tables 2, 3). The enrichment defined topoisomerases and histone deacetylases (HDAC) as prime targets for co-inhibition in both cell lines. A third prominent group of hit candidates comprised inhibitors targeting mitotic cell cycle kinases, including AURKA, AURKB, CDK1, PLK1, and WEE1 (17% of total hits in U-2932; 12% in RIVA).

**Table 2:** Hit candidate list from screen in U-2932 cells.

**U-2932**		
**Drug name**	**Target**	**Bliss score**
**10 nM**		
Mitoxantrone Hydrochloride	Topo	0.39
Idarubicin HCl	Topo	0.33
Doxorubicin (Adriamycin)	Topo	0.32
Epirubicin Hydrochloride	Topo	0.25
Flutamide (Eulexin)	Antiandrogen drug	0.16
Barasertib (AZD1152-HQPA)	Aurora	0.15
Daunorubicin HCl (Daunomycin HCl)	DNA	0.19
Oxaliplatin (Eloxatin)	DNA	0.17
Paclitaxel (Taxol)	Microtubule	0.30
Triptolide	NF-κB inhibitor	0.26
Elesclomol	Oxidative stress inducer	0.43
BX-795	PDK1	0.20
PF-3845	FAAH	0.18
Panobinostat	HDAC	0.07
**1** **μM**		
Danusertib (PHA-739358)	Aurora	0.28
VX-680 (MK-0457, Tozasertib)	Aurora	0.19
AMG 900	Aurora	0.18
MLN8237 (Alisertib)	Aurora	0.14
SNS-314	Aurora	0.12
Ponatinib (AP24534)	Abl, PDGFRα, VEGFR2, FGFR1, and Src	0.39
Dasatinib (BMS-354825)	Abl, Src, and c-Kit	0.31
Salinomycin (Procoxacin)	Anti-bacterial	0.09
PCI-32765 (Ibrutinib)	Btk	0.18
PHA-793887	CDK2, CDK5, and CDK7	0.17
JNJ-7706621	pan-CDK	0.39
LY2603618 (IC-83)	Chk1	0.16
Cytarabine	DNA	0.57
Bleomycin sulfate	DNA	0.20
Oxaliplatin (Eloxatin)	DNA	0.14
Decitabine	DNA methyltransferase	0.37
NU7441(KU-57788)	DNA-PK	0.23
SMER 3	E3 ubiquitin ligase	0.34
BIX 01294	G9a histone methyltransferase	0.16
Oxamflatin	HDAC	0.77
Vorinostat (SAHA)	HDAC	0.22
Entinostat (MS-275, SNDX-275)	HDAC	0.22
Mocetinostat (MGCD0103)	HDAC	0.12
TPCA-1	IKK-2	0.18
Pomalidomide	Inhibits LPS-induced TNF-α release	0.15
BI 78D3	JNK	0.35
Mycophenolate mofetil (CellCept)	Monophosphate dehydrogenase I/II	0.52
Sotrastaurin (AEB071)	pan-PKC	0.13
Crenolanib (CP-868596)	PDGFRα/β	0.30
BX-795	PDK1	0.13
GDC-0941	PI3Kα/δ	0.43
PIK-93	PI4KIIIβ	0.18
LY 333531	Protein kinase C	0.25
Pentostatin	Purine analog	0.14
GSK 269962	Rho-associated protein kinase	0.19
GSK 650394	Serum- and glucocorticoid-regulated kinase-1	0.18
SANT-2	Smoothened antagonist	0.19
SKI II	SphK	0.19
Bay 11-7085	TNFα-induced IκBα phosphorylation	0.23
Etoposide (VP-16)	Topo	0.31
Banoxantrone dihydrochloride	Topo	0.19
AZ 23	Trk	0.16
Tivozanib (AV-951)	VEGFR1/2/3	0.14
MK-1775	Wee1	0.38
PD 166285	Wee1/Chk1	0.86

*Color-shading indicates increasing Bliss score from yellow to dark green*.

**Table 3:** Hit candidate list from screen in RIVA cells.

**RIVA**		
**Drug name**	**Target**	**Bliss score**
**10 nM**		
Panobinostat	HDAC	0.27
Daunorubicin HCl (Daunomycin HCl)	DNA	0.22
BX-795	PDK1	0.12
GSK461364	Plk1	0.20
Doxorubicin (Adriamycin)	Topo	0.29
Mitoxantrone Hydrochloride	Topo	0.23
Idarubicin HCl	Topo	0.21
Epirubicin Hydrochloride	Topo	0.20
**100 nM**		
PCI-24781	HDAC	0.40
SB939 (Pracinostat)	HDAC	0.25
Trichostatin A (TSA)	HDAC	0.21
CUDC-101	HDAC	0.19
Belinostat (PXD101)	HDAC	0.15
Mocetinostat (MGCD0103)	HDAC	0.12
MLN8237 (Alisertib)	Aurora	0.13
Cytarabine	DNA	0.25
Mycophenolic (Mycophenolate)	IMP dehydrogenase I/II	0.12
GW3965 HCl	LXR	0.21
Cladribine	Nucleoside analog	0.24
JNJ-7706621	Pan-CDK	0.13
AG14361	PARP1	0.14
MLN2238	Proteasome	0.12
Clofarabine	Ribonucleotide reductase	0.21
Toremifene Citrate (Fareston. Acapodene)	SERM	0.19
Etoposide (VP-16)	Topo	0.27
Irinotecan HCl Trihydrate (Campto)	Topo	0.20
Doxorubicin (Adriamycin)	Topo	0.11
Epirubicin Hydrochloride	Topo	0.14

*Color-shading indicates increasing Bliss score from yellow to dark green*.

Since both topoisomerase inhibitors, such as doxorubicin or etoposide, and HDAC inhibitors are known to cause direct or indirect DNA damage, respectively, it is likely they might overcome resistance by potentiating the level of DNA damage in ^177^Lu-RIT-targeted cells (24–26). Therefore, we focused on the third group of compounds, the mitotic kinase inhibitors, that affect kinases with critical functions for both mitotic entry and exit, and have a role in termination of the DNA damage-induced G_2_-checkpoint (27–29). In particular we further explored the combination of ^177^Lu-lilotomab satetraxetan with the dual CDK1/2-Aurora A/B inhibitor JNJ-7706621, the Aurora A inhibitor alisertib (MLN8237), and the Plk1 inhibitor GSK461364, respectively. JNJ-7706621 and alisertib were hit candidates in both cell lines. GSK461364 did not score as a hit candidate in U-2932 cells only due to its high potency as a mono-therapy.

### JNJ-7706621 Synergistically Reduces Viability of DLBCL When Combined With ^177^Lu-Lilotomab Satetraxetan

To investigate the validity of the synergism observed following ^177^Lu-Lilotomab satetraxetan with the effect of the cell cycle kinase inhibitors in resistant cell lines, U-2932 cells were treated or not with ^177^Lu-lilotomab satetraxetan (0.5, 1, and 2 μg/ml) for 18 h, washed and seeded onto 384-well plates pre-printed with 11-step gradients of JNJ-7706621, alisertib, and GSK461364 ranging from 0 to 1,280 nM. Similar to the primary screen, viability was measured by RealTimeGlo. Dose-response profiles were recorded at day 5 and Combination Indexes (CI) calculated to test for a synergistic interaction of ^177^Lu-Lilotomab satetraxetan-inhibitor combinations (Chou-Thalaly; CompuSyn software). CI values were calculated for combinations within the minimum and maximum effect range of mono-treatment of each inhibitor.

**Figure 4:**
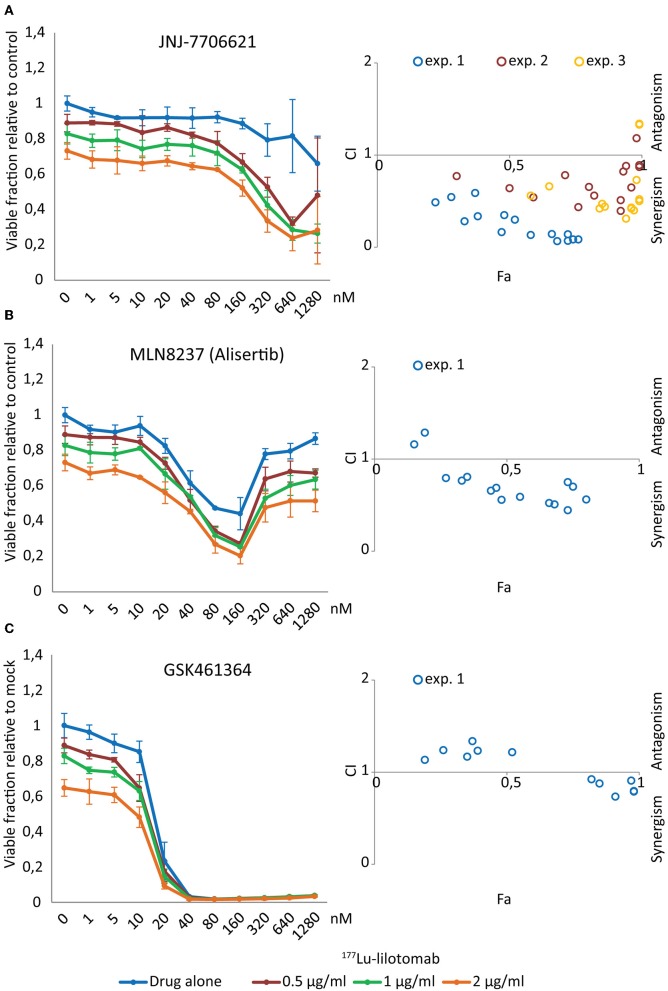
JNJ-7706621 synergistically reduces viability of U-2932 cells when combined with ^177^Lu-lilotomab satetraxetan. U-2932 cells were treated with 0.5, 1, and 2 μg/mL ^177^Lu-lilotomab satetraxetan as in Figure 1 and seeded in 384-well plates pre-printed with JNJ-7706621, Alisertib, and GSK461364 in an 11 step concentration gradient (0–1,280 nM). Viability was measured as in Figure 1B. In left panels the fraction of viable cells relative to untreated control at day 5 are plotted for cells exposed to single treatment or combinations for each drug dose (blue: drug alone, red: drug combined with 0.5 μg/mL ^177^Lu-lilotomab satetraxetan, green: drug combined with 1 μg/mL ^177^Lu-lilotomab satetraxetan and orange: drug combined with 2 μg/mL ^177^Lu-lilotomab satetraxetan) **(A)** JNJ-7706621, **(B)** Alisertib, and **(C)** GSK461364. Right panels show Fa/CIs plots obtained by the Chou-Talaly method using the CompuSyn software. The fraction affected (Fa) is the fraction of non-viable cells relative to untreated control. For each combination with a specific Fa value the CI indicates whether the combined treatment is synergistic (<1) or antagonistic (>1). In blue are data from the same experiment as that shown in left panels, whereas red and yellow circles represent two independent experiments performed in U-2932 with JNJ-7706621. In experiment 2, 100, 266, 707, 1,880, and 5,000 nM of JNJ-7706621 were combined with the same doses of ^177^Lu-lilotomab satetraxetan as in experiment 1, and in experiment 3 the doses of JNJ-7706621 were; 200, 532, 1,410, 3,760, and 10,000 nM. Error bars: STDEV of triplicate samples.

Figure 4 shows dose-response curves for treatment with either drug alone or in combination with different doses of ^177^Lu-lilotomab satetraxetan. The anti-proliferative effect of the dual CDK1/2-AURKA/B inhibitor JNJ-7706621 was moderate, even at high doses (Figure 4A). However, and confirming the primary screen results, the combination with ^177^Lu-lilotomab satetraxetan led to a greater reduction in the fraction of viable cells than each treatment alone. Synergism (CI <1) was observed for all tested combinations (blue circles in Fa/CI plots). The AURKA inhibitor alisertib had a bi-phasic dose-response profile, with a decreasing anti-proliferative effect at doses above 160 nM (Figure 4B) and synergism was observed within a range of 10–160 nM. U-2932 cells were highly sensitive to treatment with the PLK1 inhibitor GSK461364 with a near complete growth inhibition obtained at 20 nM (Figure 4C). At lower doses the sensitivity was greatly reduced, and when combined with ^177^Lu-lilotomab satetraxetan growth inhibition was, however modestly, potentiated. Weak synergism (CI 0.75–0.95) was observed only at GSK461364 concentrations near the maximum effect (20 and 40 nM; Fa close to 1). Similar CI index profiles were obtained in RIVA cells (Supplementary Figure 2). The results of the validation screen identified the dual CDK1/2 and AURKA/B kinase inhibitor JNJ-7706621 as the best hit candidate. We hence controlled first for target-specificity/activity for inhibition of CDK1 and AURKB (Supplementary Figure 3). Synergism with ^177^Lu-lilotomab satetraxetan was then confirmed in two additional combination experiments in U-2932 cells covering the range of 100–10,000 nM JNJ-7706621 (Figure 4A, yellow and red circles).

### The Dual CDK1/2 and AURKA/B Inhibitor JNJ-7706621 Synergizes With CD37-Targeted RIT by Potentiating Mitotic Slippage and Apoptotic Cell Death

**Figure 5:**
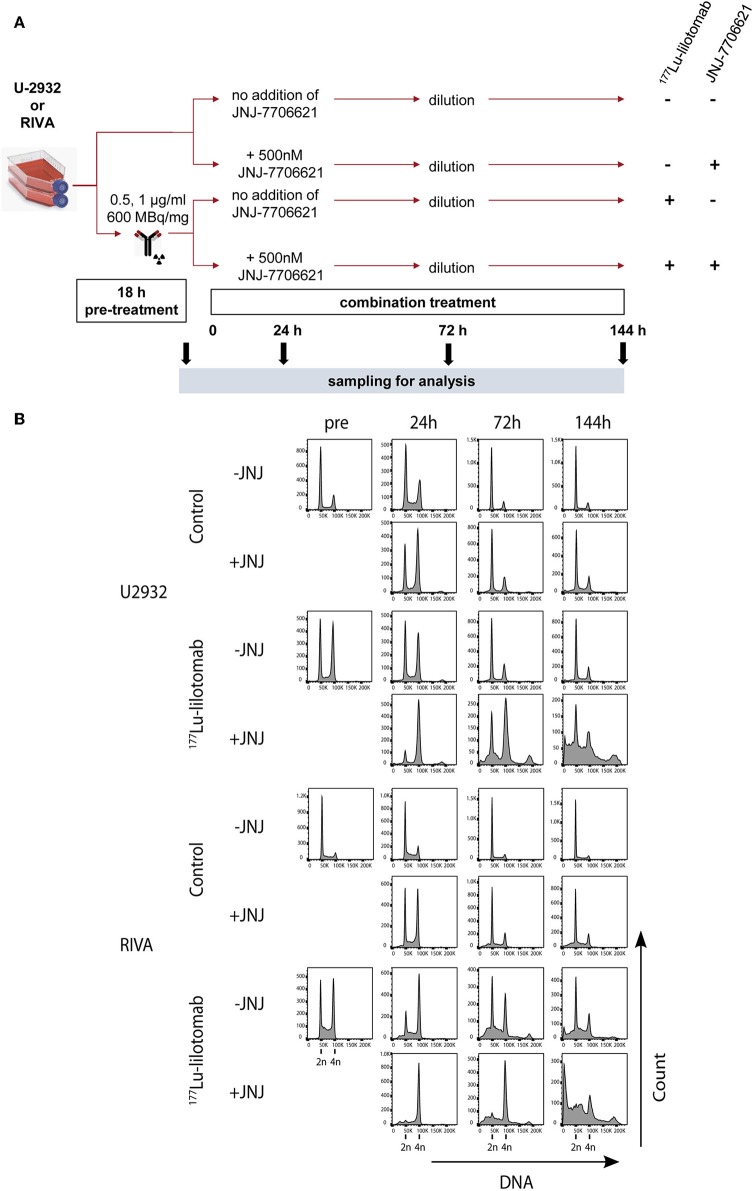
JNJ-7706621 leads to extended G2/M arrest and endoreduplication in ^177^Lu-lilotomab satetraxetan treated cells. **(A)** Outline of experiment. U-2932 and RIVA cells were treated as in Figure 1 with indicated doses of ^177^Lu-lilotomab satetraxetan. JNJ-7706621 was added to treated and untreated cells to a final concentration of 500 nM. Samples were harvested for flow analysis before (pre) and 24, 72, and 144 h after adding inhibitor. After harvesting 72 h sample cultures were replenished with medium containing inhibitor. Before fixation, cells were stained with Pacific Blue in order to discriminate between live and dead cells. FxCycleFar Red was used to stain DNA. **(B)** DNA histograms showing cell cycle distribution in U-2932 (upper panels) and RIVA (lower panels). Data shown are representative of two independent experiments.

The validation experiments confirmed a synergistic drug interaction of JNJ-7706621 and ^177^Lu-lilotomab satetraxetan in the inhibition of proliferation of U-2932 and RIVA cells, as assessed by the decreased capacity in reducing the RealTimeGlo substrate. CD37-targeted RIT induced DNA damage and inhibition of CDK1/2 and AURKA/B kinases by JNJ-7706621 are independently of each other expected to result in cell cycle progression defects. For instance, in mitotic shake-off synchronized HeLa cells, inhibition of CDK1/2 and AURKA/B by JNJ-7706621 at a final concentration of 1–3 μM was reported to delay exit from G_1_, to arrest cells at G_2_/M, and to induce endoreduplication (30). We thus investigated the effects of mono- and combination therapy on cell cycle progression in U-2932 and RIVA cells (Figure 5). Cells were treated with or without ^177^Lu-lilotomab satetraxetan (18 h; U-2932: 1 μg/ml, RIVA 0.5 μg/ml), washed and treated with or without 500 nM JNJ-7706621, a concentration at which strong synergism was observed in both cell lines (Figure 4 and Supplementary Figure 2).

**Figure 6:**
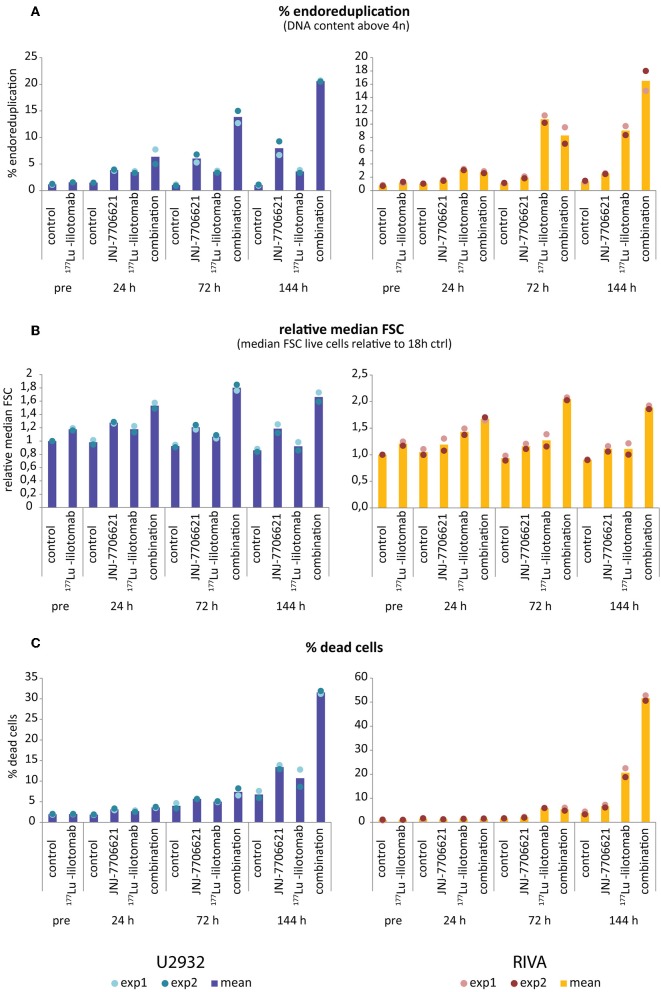
Increase in >4n population and cell size precedes cell death upon combined treatment with JNJ-7706621 and ^177^Lu-lilotomab satetraxetan. Results are from the same experiment as in Figure 5 and a parallel experiment. **(A)** Percentage of cells with DNA content above 4n. **(B)** Median forward scatter (FSC) of live cells (gated on Pacific Blue channel), all values are relative to untreated control of 18 h pre-treatment. **(C)** Percentage of dead cells as measured by Pacific blue staining. Bar diagrams show mean of two separate experiments, with individual data indicated with dots. Left panels: U-2932, right panels: RIVA.

**Figure 7:**
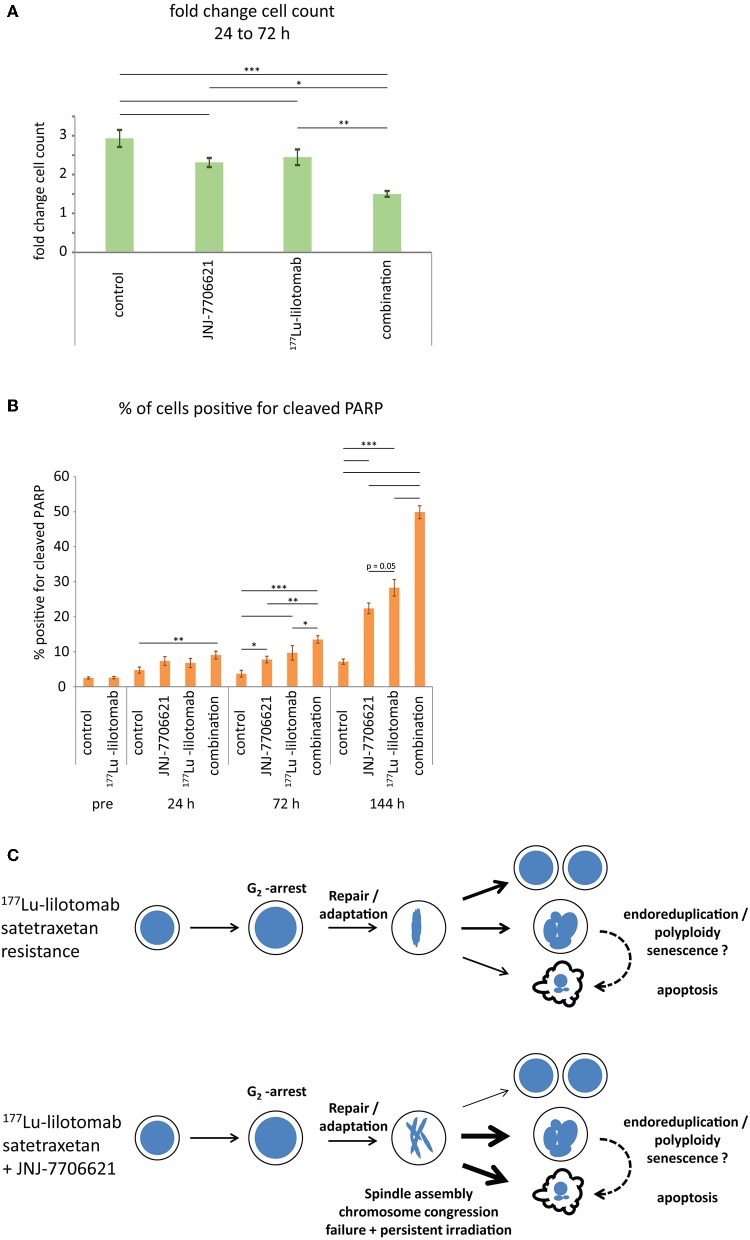
Combined treatment with ^177^Lu-lilotomab satetraxetan and JNJ-7706621 induces growth delay and apoptotic death. Cells were treated as in Figure 5. **(A)** Bar diagram showing relative cell growth of cells between 24 and 72 h after treatment (*n* = 4; error bars represent standard error of the mean). **(B)** Bar diagram showing percentage of cells positive for cleaved PARP (*n* = 4; error bars represent standard error of mean (*n* = 4). **(A,B)** Statistical significance in differences between treatment groups were tested by One Way ANOVA: ^*^p < 0.05, ^**^p < 0.01, ^***^p < 0.001. **(C)** Model: treatment with ^177^Lu-lilotomab satetraxetan leads to DNA-damage induced G_2_ arrest and apoptotic cell death. Cells resistant to treatment adapt and recover from the arrest. Inhibition of CDK1 and AURKA/B interferes with bipolar- and mid-spindle assembly, causing chromosome congression and cytokinesis defects. Combined treatment with JNJ-7706621 and ^177^Lu-lilotomab satetraxetan reverses resistance likely by potentiating the effect of persistent radiation due to extended residence time in and failure of mitosis, the cell cycle phase in which repair capacity is low.

Samples were taken immediately after treatment with ^177^Lu-lilotomab satetraxetan and at 24, 72, and 144 h after inhibitor addition, and DNA content and cell death assessed by flow cytometry (Figure 5A). Medium was replenished (with or without inhibitor) at 72 h to allow for optimal growth conditions throughout the observation period. Treatment with JNJ-7706621 alone induced a prominent but transient accumulation of cells in G_2_/M-phase (4n DNA content) after 24 h of treatment in both cell lines (Figure 5B). Similarly, pre-treatment with ^177^Lu lilotomab satetraxetan alone also induced a prominent but transient accumulation of cells in G_2_/M-phase in both cell lines. The addition of JNJ-7706621 to ^177^Lu-lilotomab satetraxetan treated cells strongly reduced the fraction of cells in G_1_ phase (2n) at the 24 h time time-point and at later times strongly increased appearance of single cells with <2n or >4n DNA content, indicative of cell death and endoreduplication/cytokinesis failure, respectively. We therefore quantitatively assessed the fraction of endoreduplication (>4n DNA content), cell death (Pacific blue uptake), as well as cell size (median forward scatter) in both cell lines in two independent experiments (Figure 6 and Supplementary Figures 4, 5). Combination treatment strongly induced the formation of endoreduplicating cells (20- and 12-fold increase in U-2932 and RIVA, respectively; Bliss CI U-2932 = 0.55, RIVA = 0.69; [CI = ((E_RIT_ + E_JNJ_)–E_RIT_ × E_JNJ_)/E_[RIT+JNJ]_]), which was accompanied by a 2-fold increase in relative cell size, compared to untreated and mono-treated cells. Visual inspection of cells by microscopy confirmed this result, revealing prominent occurrence of multinucleated cells and increased DNA content (Supplementary Figure 6). Most importantly, combination treatment led to a 5- and 13-fold increase in cell death compared to untreated cells at the 144 h time point in U-2932 (CI = 0.72) and RIVA (CI = 0.50), respectively. To further explore the mechanistic details behind the anti-tumor activity of JNJ-7706621 and ^177^Lu-litomab satetraxetan combination treatment, we used the same experimental setup as described in Figure 5 but now monitored PARP cleavage, a late apoptotic event, and cell growth (Figure 7). In accordance with our RealTime-Glo data we found that cells exposed to the combination showed a significantly reduced growth rate between 24 and 72 h post-treatment as compared to control cells and cells receiving monotherapy (Figure 7A). The fraction of cells in late apoptosis (cleaved PARP positive cells), was at all tested time-points significantly increased in cells receiving combination therapy as compared to control cells, reaching about 50% after 144 h (Figure 7B). Induction of apoptosis was also significantly increased in cells receiving single agent therapy at 72 and 144 h time points compared to control. At 144 h, 22% of JNJ-7706621 and 28% ^177^Lu-litomab satetraxetan treated cells were determined as late apoptotic, indicating an additive effect of the combination treatment. Taken together, these results suggest that the synergistic anti-proliferative effect of the combination of JNJ-7706621 and ^177^Lu-litomab satetraxetan is a consequence of JNJ-7706621 mediated mitotic infidelity of cells which have over-come the ^177^Lu-litomab satetraxetan induced G_2_-arrest, leading to incompatibility with proliferation and to excessive cell death by apoptosis (Figure 7C).

## Discussion

Targeted radionuclide delivery for DNA damaging radiation by means of antibody-conjugates has shown promising efficacy in clinical studies in the treatment of hematological cancers. ^90^Y-Ibriumomab and ^131^I-tositumomab have demonstrated significant activity in indolent relapsed/refractory NHL. ^177^Lu-lilotomab satetraxetan is emerging as a potential treatment option for patients with rituximab resistant relapsed/refractory FL as well as R-CHOP resistant (and ASCT in-eligible) DLBCL. Here, we identified two ABC-DLBCL cell lines, U-2932 and RIVA, with primary resistance to CD37-targeting ^177^Lu-lilotomab satetraxetan treatment, derived from DE ABC-DLBCL with inactive TP53. Subsequently, we used these cell lines to screen for compounds able to prevent the resistance to RIT and we identified and characterized the dual-specific CDK1/2 and AURKA/B kinase inhibitor JNJ-7706621, alongside topoisomerase and HDAC inhibitors. Alike other RITs ^177^Lu-lilotomab satetraxetan is likely to induce a DNA damage response mediated cell cycle G_2_ arrest that resistant cells are required to overcome or adapt to. Our findings may thus be of particular importance as G_1_ arrest abrogating subclonal *TP53* mutations were recently found to be predictive of PFS in FL patients treated with CD20-targeting RIT-CHOP (^131^I Tositumomab), but not R-CHOP (31).

U-2932 and RIVA are notoriously treatment resistant cell line models of ABC-DLBCL, including radiation- and chemotherapy (32, 33). Importantly, loss of or decreased binding to CD37 was excludable as an underlying cause of resistance to CD37-targeting RIT. The unbiased screening for anti-cancer compounds, which in combination with ^177^Lu-lilotomab satetraxetan synergistically impair proliferation of these cell lines identified three major classes of compounds that may be utilizable to overcome RIT-resistance: topoisomerase inhibitors, HDAC inhibitors, and inhibitors of mitotic cell cycle kinases. Since we tested only a limited set of concentrations in a sequential approach following exposure to RIT, our study might have missed additional compounds that might be beneficial in combination at different concentrations or when given before or concomitantly with RIT, although radiation is persistently delivered through binding of the radioisotope conjugated antibody to CD37.

Interestingly, all Topoisomerase and all HDAC inhibitors within the compound library scored in at least one cell line. Topoisomerase inhibitors, such as doxorubicin and etoposide, are essential constituents of standard and salvage chemotherapy regimens (R-CHOP and R-ICE/R-DHAP) for lymphoma treatment and may synergize with ^177^Lu-lilotomab satetraxetan by exaggerating the cumulative DNA damage beyond the cell's capacity of repair. HDAC inhibitors have been shown to lead to excessive DNA damage in cancer cells (24), but other downstream aspects within the pleiotropic effects of epigenetic modification may contribute to or be essential for the observed synergistic interaction. They have shown anti-tumor activity especially in T-cell lymphomas and are currently under evaluation in different combination regimens for r/r-DLBCL (34). Hence, both classes of compounds might not only potentiate the amount of DNA damage in ^177^Lu-RIT-targeted cancer cells but also confer off-targeted DNA damage in untransformed cells due to their systemic administration. We focused on the third enriched group composed of mitotic cell cycle kinases inhibitors, which represent a secondary main (independent) target different from induction of DNA damage. Their inhibition ultimately interferes with balanced segregation of chromosomes as well as separation of daughter cells, rather than the direct DNA damage induction of replicating cells (27–29). Mitotic kinase inhibitors, including the hit candidate MLN8237 (alisertib), are currently in clinical trials for B cell lymphomas, e.g., Friedberg et al. (35) and Kelly et al. (36) (NCT00807495; NCT00697346). The dual-specific inhibitor of CDK1/2 and AURA/B kinases, JNJ-7706621, outperformed the PLK1 inhibitor GSK461364 and the AURKA inhibitor alisertib in the validation screen on the basis of strong synergism within a broad concentration range, at which mono-treatment with JNJ-7706621 showed little to no anti-proliferative efficacy. In contrast, synergism with PLK1 inhibition was only confirmed at concentrations near the maximum effect of the highly potent GSK461364 inhibitor itself. The high efficacy of GSK461364 alone may suggest its applicability (or that of equivalent PLK1 inhibitors) in mono-therapy for treatment of CD37-targeting RIT-resistant DLBCL or aggressive ABC-DLBCL. Clearly, further pre-clinical evaluation will be required to support this hypothesis. GSK461364 showed dose-related anti-tumor activity in a phase 1 study, but concomitantly lead to a high incidence rate of venous thrombotic emboli (37). Furthermore, acquired resistance due to up-regulation of ATP-binding cassette drug transporters has been reported for GSK461364 and alternative PLK1 inhibitors, including BI2536 and the clinically most advanced BI6727/Volasertib, potentially favoring combination over monotherapy strategies (38–40). Synergism of ^177^Lu-lilotomab satetraxetan with alisertib was less strong than with JNJ-7706621, and confined to a narrow concentration window, since alisertib demonstrated a loss of efficacy at concentrations above 360 nM. The latter may be a concentration-dependent compound de-activating artifact of formulation in DMSO or a consequence of an antagonistic interaction with a secondary “off-target” inhibited enzyme. *AURKA (STK15)* is a driver and essential gene in DLBCL (41) and the identification of several other AURKA inhibitors in our screening campaign (not validated here) are supportive of further investigations. Pre-clinical and clinical investigations of alisertib in DLBCL treatment strongly suggest the need for combination drug partners, since re-commitment of treatment induced senescent aneuploid cells to cell cycle progression and low mitotic index in primary tumors are apparent causes of treatment resistance (29, 42–44). Hence, a more elaborate analysis of ^177^Lu-lilotomab satetraxetan with a panel of AURKA inhibitors may prove valuable. This is strongly supported by the robust synergistic interaction of CD37-targeting RIT with JNJ-7706621 reported here. Dual-specificity and activity of JNJ-7706621 against CDK1/2 and AURA/B kinases was shown in TP53 proficient and deficient cell lines (45) and observed in U-2932 and RIVA cells. This inhibitor has shown anti-tumor activity in mouse xenograft models of solid tumors, but not been tested in clinical trials (30, 46). Of note, pre-clinical studies of JNJ-7706621 activity investigated its effect in the μM range, where anti-proliferative activity in monotreatment is evident (30, 45). Here, we showed that even sub-μM concentrations of JNJ-7706621 were sufficiently strong enough to synergize with ^177^Lu-lilotomab satetraxetan, reducing the potential toxicity-risk of this compound and concomitantly lowering its required active concentration. The *in vivo* synergistic interaction of JNJ-7706621 and CD37-targeting RIT remain, however, to be proven.

Kinetic studies of the effect of mono- and combination therapy of U-2932 and RIVA cells with JNJ-7706621 and ^177^Lu-lilotomab satetraxetan are suggestive of a model (Figure 7) in which radiation damage induced G_2_-arrested lymphoma cells eventually enter mitosis (repair or escape) and mitotic entry, progression and exit are impaired by JNJ-7706621 mediated inhibition of CDK1/2 and AURKA/B. DNA damage in mitosis is a known driver of chromosomal instabilities (47, 48). The extended residence-time of cells in mitosis due to chromosome condensation and congression defects as well as spindle and mid-spindle assembly failure is pivotal for the increased sensitivity to persistent ^177^Lu-lilotomab satetraxetan deposited DNA damage, ultimately promoting cytokinesis failure (multinucleation, aneuploidy, increased cell size) and cell death by apoptosis.

In conclusion, CD37-targeting ^177^Lu-lilotomab satetraxetan RIT showed activity in several ABC-DLBCL lymphoma cell lines. CD37-independent RIT-resistance was identified in two cell lines representative of aggressive DE ABC-DLBCLs with inactive TP53, and reversed by subsequent inhibition of CDK1/2 and AURKA/B by JNJ-7706621. These findings are of specific relevance for ongoing clinical trials of ^177^Lu-lilotomab satetraxetan in relapsed, ASCT-non-eligible DLBCL, and may also be more generally applicable to other ^177^Lu-based RITs and alternative radionuclide utilizing targeted therapies. Future pre-clinical investigations are required to elucidate the potential application of CDK1/2 and AURKA/B inhibitors as a strategy to revert RIT resistance in *TP53* deficient cancers.

## Materials and Methods

### Cells and Reagents

ABC-DLBCL cell lines were maintained in RPMI 1640-GlutaMAX medium (Gibco 61870-044) supplemented with 15% fetal bovine serum (Biowest S181B-050) and 1% penicillin-streptomycin (Gibco 15140-122) at 37°C in a humidified atmosphere containing 5% CO_2_. Cell lines were obtained as previously described (21) and their identity was authenticated by short tandem repeat DNA profiling (IDEXX BioResearch, Ludwigsburg, Germany). Real Time Glo was from Promega (G9713). The Selleck Cambridge Cancer Compound library was obtained from and printed onto 384-well plates [384 well, PS, F-bottom, μclear, white, lid, sterile, Greiner Bio-One 781098 (82050-076)] by the High-Throughput Chemical Biology Screening Platform at the Center for Molecular Medicine Norway (NCMM). Antibodies used were: rabbit-anti-phospho-BRCA2(Ser3291) (AB9986 Millipore), rabbit-anti-Cleaved-PARP (Asp214) Alexa647-conjugate (#6987, Cell Signaling), rabbit-anti-phospho-histone 3-Ser10 (06-570, Millipore), Alexa488 anti-rabbit (A21206, Life Technologies), and Alexa647 donkey-anti-mouse (Jackson ImmunoResearch, UK). The DNA stain FxCycle™ Far Red (200 nM FxCycle and 0.1mg/ml RNase A) (Thermo Fisher Scientific) was used together with Pacific Blue (Thermo Fisher Scientific, P10163) staining, and Hoechst 33258 (Sigma-Aldrich) (1.5 μg/ml) in other experiments.

### Labeling of Antibody With ^177^Lu

The chelator (p-SCN-Bn-DOTA, Macrocyclics, TX, USA) was dissolved in 0.005 M HCl, added to the HH1-DOTA (lilotomab) in a 6:1 ratio and pH-adjusted to ~8.5 using carbonate buffer. After 45 min of incubation at 37°C, the reaction was stopped by the addition of 50 μL per mg of Ab of 0.2 mol/L glycine solution. To remove free p-SCN-Bn-DOTA, the conjugated antibody was washed using Vivaspin 20 centrifuge tubes (Sartorius Stedim Biotech, Göttingen Germany) 4–5 times with NaCl 0.9%. Before labeling with ^177^Lu, the pH was adjusted to 5.3 ± 0.3 using 0.25 mol/L ammonium acetate buffer. Between 120 and 220 MBq of ^177^Lu (ITG, Garching, Germany) was added to 1 mg of satetraxetan-Ab and incubated for 15–30 min at 37°C. The radiochemical purity (RCP) of the conjugate was evaluated using instant thin-layer chromatography. If RCP was below 95% the conjugate was purified by elution through a Sephadex G-25 PD-10 column (GE Healthcare Bio-Sciences AB, Uppsala, Sweden).

### Treatment With ^177^Lu-Lilotomab Satetraxetan (Betalutin)

Cells were treated in 6-well plates (2.5 × 10^6^ cells/mL) without shaking for 18 h with Betalutin (specific activity of ~600 Mbq/mg) at a final concentration of 1 μg/ml for U2932 and 0.5 μg/ml for RIVA (or as otherwise specified). After treatment, PBS was added to the cells, and the cells pelleted. Cells were first resuspended in 1 ml PBS, then washed twice in PBS and finally diluted in growth medium to desired concentration for the assay applied.

For the initial screening of ABC-DLBCL cell lines cells were incubated in deep well plates (Nunc™ 96-Well Polypropylene DeepWell Storage Plates from Thermo Scientific) with 12 different concentrations of ^177^Lu-lilotomab satetraxetan, including a control with no treatment, for 18–20 h with shaking. Cells were washed three times with PBS using a plate washer (ELx405 Select Deep Well Washer from BioTek) and seeded in 96-well plates in 0.2 ml medium and incubated at 37°C/5% CO_2_. Fifty microliter of fresh medium was added after 72 h. The cytotoxic effect was measured at 144 h using the CyQuant NF Cell Proliferation Assay kit (ThermoFischer) for Ascent FL multiplate reader (ThermoFischer. IC50 were calculated using Prism (GraphPad) and clustering analysis performed in J-Express Pro (49).

### Combinatorial Drug Screen

Cells treated with ^177^Lu-lilotomab satetraxetan and untreated control cells were seeded onto 384-well plates pre-printed with the 384-compound Cambridge Cancer Compound library sourced from SelleckChem. The library was divided onto two plates and a total of 48 no-drug controls were included per plate. Cell seeding densities were 120 000 cells/mL, and a volume of 25 μL was seeded in each well-giving 3,000 cells/well. The drug library was screened at two concentrations for each cell line (10/100 nM for RIVA and 10/1,000 nM for U-2932). Three days after seeding, 25 μL of diluted NanoLuc® luciferase and MT Cell Viability Substrate (RealTime-Glo) was added to each well. Cells were incubated with the reaction mix for 1 h at 37°C before measuring luminescence in a Tecan Spark multimode microplate reader, with integration time set to 1 s. Luminescence readings were repeated each day for three consecutive days. A microplate sample processor (Precision XS, BioTek) was used to facilitate the cell seeding and dispensing of RealTimeGlo reagent. Hit candidates were identified using the Bliss Independence test for synergy. The effect of each drug alone (Fa) at each concentration was calculated as the fraction of dead cells as compared to control cells

$$\begin{array}{l} Fa=1-\left(\frac{RLU_{drug}}{averageRLU_{control}}\right) \end{array}$$

a similar calculation was performed for the average effect of ^177^Lu-lilotomab satetraxetan alone ($\bar{Fb}$)

$$\begin{array}{l} \bar{Fb}=1-\left(\frac{averageRLU_{177Lu-lilotomab}}{averageRLU_{control}}\right) \end{array}$$

Through the following equations we found the expected additive effect (E) and the measured effect (M) of the combination of drug + ^177^Lu-lilotomab satetraxetan:

$$\begin{array}{lll} E & = & Fa+\bar{Fb}-Fa*\bar{Fb}\\ M & = & 1-\left(\frac{RLU_{combination}}{averageRLU_{control}}\right) \end{array}$$

The Bliss score was defined as:

$$\begin{array}{l} Bliss\;score=\frac{M-E}{1-Fa} \end{array}$$

The normalization to the fraction of viable cells in samples treated with drug alone (1-Fa in equation above) was carried out to compensate for the reduced population of cells that could be affected by combined treatment with ^177^Lu-lilotomab satetraxetan. The standard deviation for the effect of ^177^Lu-lilotomab satetraxetan alone in the 48 control wells was calculated on each plate as follows:

$$\begin{array}{l} s=\sqrt{\sum_{i=1}^{n}\frac{{\left(Fb_{i}-\bar{Fb}\right)}^{2}}{n-1}} \end{array}$$

Drugs with a Bliss score two times higher than the standard deviation of ^177^Lu-lilotomab satetraxetan treated controls were scored as potential hits. Finally, we excluded inhibitory drugs that alone reduced viability by >90% to that of untreated control. Each plate was treated individually. In validation experiments the same procedure was used with the following modifications; Cells were treated in 12-well plates (1.2 ml per well, with 2.5 × 10^6^ cells/ml) without shaking for 18 h with ^177^Lu-lilotomab satetraxetan at final concentrations of 0.5, 1, and 2 μg/ml for U2932 and 0.25, 0.5, and 1 μg/ml for RIVA. After treatment, cells were seeded on 384-well plates pre-printed with JNJ-7706621, Alisertib and GSK461364 in an 11 step concentration gradient (0-1280 nM). A total of 60 no-drug controls were included on each plate. Synergy of drug/^177^Lu-lilotomab satetraxetan combinations was determined by calculating Combination Indexes (CI) using the Chou-Talaly theorem (CompuSyn software) (50), where CI < 1 represents synergy. In replicates of validation experiment cells were treated with ^177^Lu-lilotomab satetraxetan in 96 deep-well plates (100 μL per well, with 2.5 × 10^6^ cells/ml) for 18 h, diluted to 40 cells/μL and 25 μL transferred to 384-well plates. Immediately after seeding a Tecan D300 Digital dispenser was used to administer drug to wells at 100, 266, 707, 1,880, and 5,000 nM (f.c. experiment 2) or 200, 532, 1,410, 3,760, and 10,000 μM (f.c experiment 3).

### Flow Cytometry

For live/dead discrimination, ^177^Lu-lilotomab satetraxetan treated cells were diluted to 500 000 cells/mL, transferred to T-25 cell culture flasks and inhibitor (JNJ-7706621) added to a final concentration of 500 nM. ^177^Lu-lilotomab satetraxetan treated and untreated control samples, with or without inhibitor, were harvested for flow cytometry analysis before and at 24, 72, and 144 h after addition of the inhibitor (Figure 5A). At 72 h, 6 mL of fresh medium w/wo inhibitor was added to allow continuous growth of the cells that were harvested at the latest timepoint (144 h). Before fixation, cell pellets were resuspended in 200 μL PBS with 18 ng/μL of Pacific Blue and incubated at 4°C for 20 min for live/dead discrimination. One milliliter of PBS was added to the samples, and cells were pelleted and resuspended in 1 mL of ice-cold 70% ethanol for fixation. Samples were stained with FxCycle Far Red for visualization of DNA, and analyzed on an LSR II flow cytometer (BD Biosciences) using FlowJo software. The same experimental setup (omitting Pacific Blue) was followed for assessment of PARP cleavage. Cells were fixed in 1.5% neutral buffered formaldehyde for 5 min at r.t, washed in PBS, and stored in Methanol (−20°C). For analysis, cells were rehydrated and stained with anti-cleaved-PARP-Asp214 and Hoechst. Cell counts in Figure 7A were determined in quadruplicates for each time-point using a Countess Automated Cell counter (Thermo Fisher). For testing efficacy of JNJ-7706621, cells were arrested in mitosis by culturing for 16 h in medium containing nocodazol (0.04 μg/ml). Still in the presence of nocodazol, cells were treated with JNJ-7706621 for 1 or 6 h at a final concentration of 250, 500, or 1,000 nM before fixation in ice-cold 70% ethanol. Samples were stained with primary antibodies against CDK and Aurora B targets phospho-BRCA2-Ser3291 and phospho-histone H3-Ser10, respectively.

CD37-expression in U-2932, RIVA and Oci-Ly10 was measured by determination of the binding capacity of cold antibody (HH1-dota). For accurate comparison of different cell lines, we included barcoding with CellTracer stain and pooled the samples from the different cell lines together in a single tube before staining with the CD37 antibody. To this, 2 × 10^e6^ cells were pelleted and resuspended in 1 ml PBS and labeled with CellTrace stain (U-2932: CFSE 1 μM f.c., RIVA: Violet 2 μM f.c., Oci-Ly10: blank) in the dark for 20 min at 37°C with gentle shaking every 3–5 min. Cells were diluted in 5 ml of pre-warmed medium. After 5 min, cells were pelleted and resuspended in 1 ml fresh medium, pooled and an additional 1 ml of medium added. Cells were kept on ice thereafter. The cell mixture was divided into four polystyrene tubes and pelleted. Primary antibody (HH1-dota) was diluted in medium and added to three of the four tubes (no antibody, 1, 2, and 20 μg). Cells were incubated on ice for 20 min, washed twice with cold PBS and resuspended in medium containing Alexa647 donkey-anti-mouse (Jackson ImmunoResearch, UK) at 1 μl/ml. After 20 min incubation on ice, cells were resuspended in 1 ml cold PBS w/1% Fetal Bovine Serum and analyzed on a LSR II flow cytometer.

### Microscopy

Cells were imaged using a CellObserver microscope system (Carl Zeiss) equipped with a 20×/0.8 PlanApo Phase 2 lens, a Hamamatsu ORCA-Flash4.0 v3 camera, a temperature controlled XL-chamber, a temperature, humidity and CO_2_ controlled stage incubator, a motorized coded X,Y-stage, a Definite Focus system and a HXP120 Metal-Halide illumination unit. Visual inspection of cells prepared for flow cytometry was conducted using 96-well flat-bottom plates (Greiner Bio-One, Kremsmünster, AUT).

## Data Availability Statement

All datasets generated for this study are included in the article/Supplementary Material.

## Author Contributions

FB, GR, JD, KM, RS, and SP contributed to the conception and design of the study. GR, KM, RG, SG, and SP performed the experiments presented in the study. Data analysis was carried out by GR, JD, RS, and SP. GR and SP wrote the first draft of the manuscript. FB, GR, JD, RS, and SP contributed to the manuscript revision. All authors read and approved the submitted version.

### Conflict of Interest

SP: Nordic Nanovector ASA: Employment, Patent. KM, RG, and JD: Nordic Nanovector ASA: Employment, Equity Ownership, Patents. GR: Patent. RS: Institutional research funds from Nordic Nanovector ASA, Patent. FB: Institutional research funds from Acerta, ADC Therapeutics, Bayer AG, Cellestia, CTI Life Sciences, EMD Serono, Helsinn, ImmunoGen, Menarini Ricerche, NEOMED Therapeutics 1, Nordic Nanovector ASA, Oncology Therapeutic Development, PIQUR Therapeutics AG; consultancy fee from Helsinn, Menarini; expert statements provided to HTG; travel grants from Amgen, Astra Zeneca, Jazz Pharmaceuticals, PIQUR Therapeutics AG. The remaining author declares that the research was conducted in the absence of any commercial or financial relationships that could be construed as a potential conflict of interest.
