# Supplementary material for: The Dual Cell Cycle Kinase Inhibitor JNJ-7706621 Reverses Resistance to CD37-Targeted Radioimmunotherapy in Activated B Cell Like Diffuse Large B Cell Lymphoma Cell Lines
Source: Front Oncol. 2019 Nov 29;9:1301. doi: 10.3389/fonc.2019.01301 (PMC6897291; doi:10.3389/fonc.2019.01301)
Supplement: Supplementary file 3 [file Data_Sheet_1.pdf]

SUPPLEMENTARY MATERIAL

Supplementary Figures Rødland et. al

Supplementary Figure 1

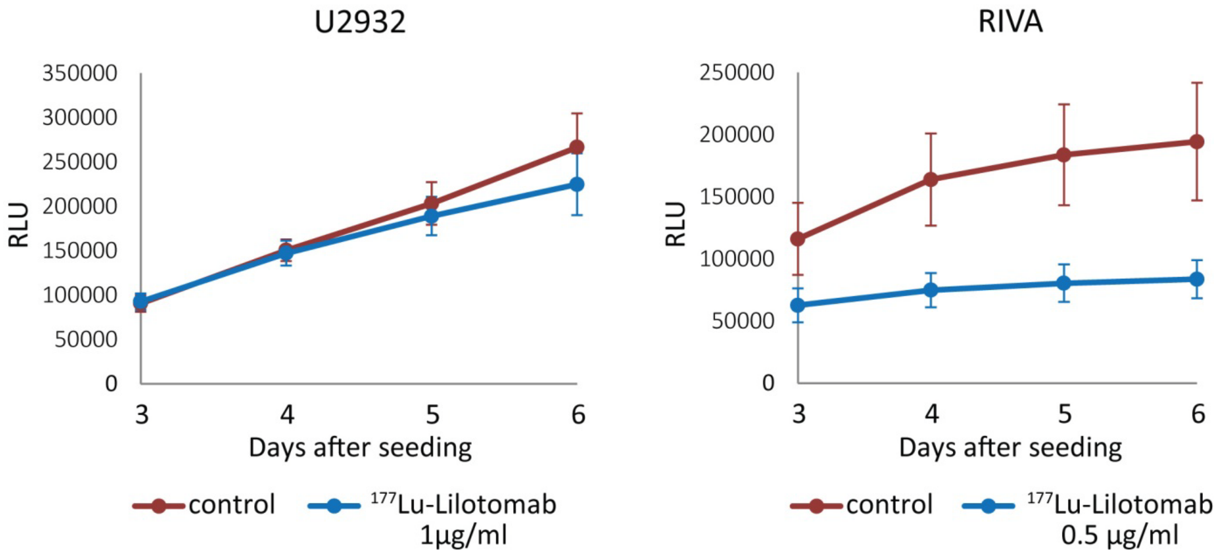

Supplementary figure 1 U-2932 and RIVA cells proliferate in the presence of ^{177}Lu-lilotomab satetraxetan

Data are from the same experiment as in Fig. 3. Relative luminescence (RLU) obtained from the RealTime-Glo assay has been plotted for each measurement. Red line represents the mean of all untreated controls from four plates, and blue line the mean of all ^{177}Lu-lilotomab satetraxetan treated controls. Error bars: STDEV (n=192).

Supplementary Figure 2

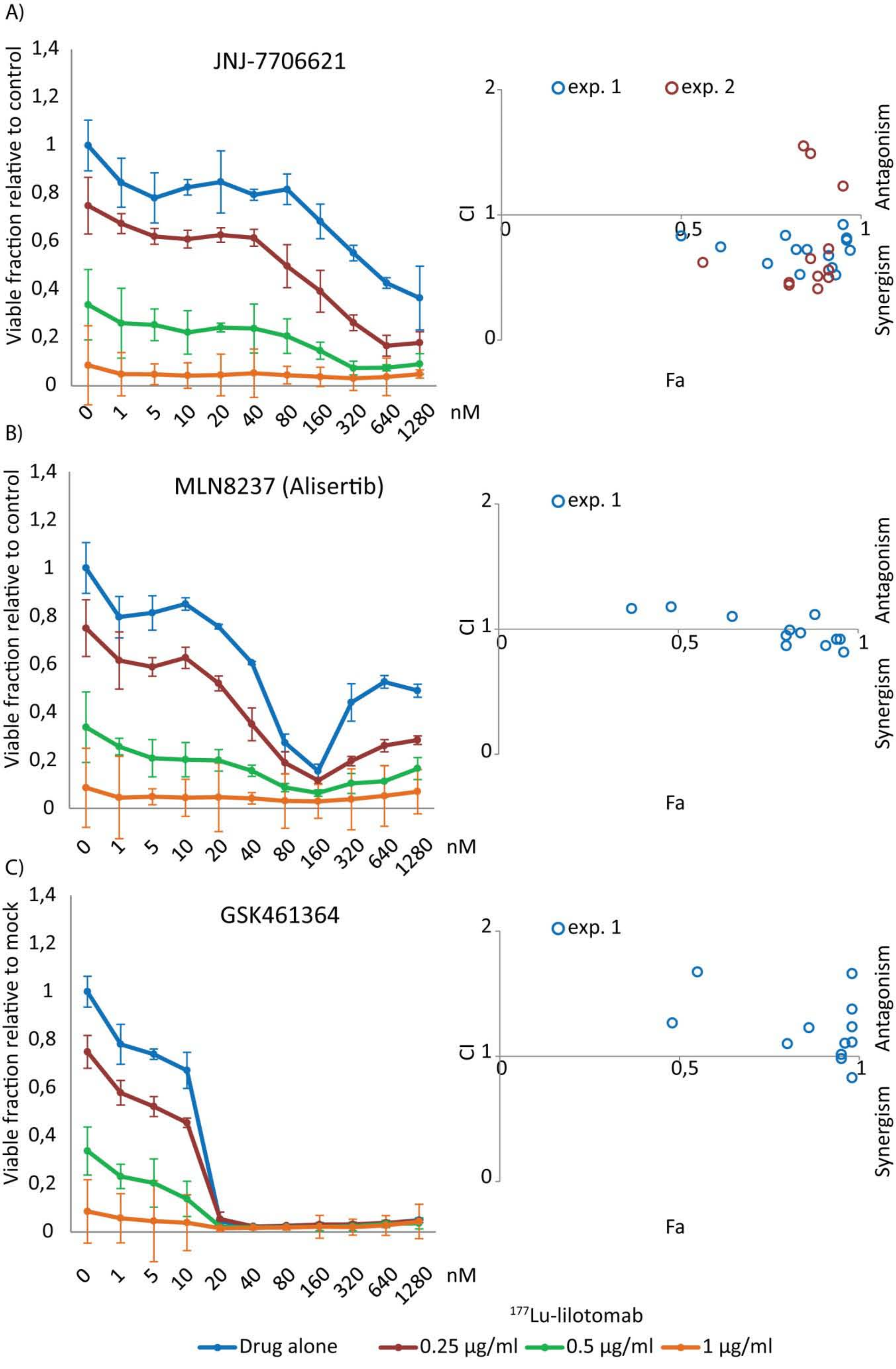

Supplementary Figure 2 JNJ-7706621 synergize with ^{177}Lu-lilotomab satetraxetan to reduce viability in RIVA cells

RIVA cells were treated with 0.25, 0.5 and 1 µg/ml ^{177}Lu-lilotomab satetraxetan and seeded in 384-well plates pre-printed with JNJ-7706621, Alisertib and GSK461364 as in Fig. 4. In left panels the fraction of viable cells relative to untreated control at day 5 are plotted for cells exposed to single treatment or combinations for each drug dose (blue: drug alone, red: drug combined with 0.25 µg/ml ^{177}Lu-lilotomab satetraxetan, green: drug combined with 0.5 µg/ml ^{177}Lu-lilotomab satetraxetan and orange: drug combined with 1 µg/ml ^{177}Lu-lilotomab satetraxetan). Right panels show Fa/CIs plots as in Fig. 4. In blue are data from the same experiment as that shown in left panels, whereas red circles represent one independent experiment with JNJ-7706621. In experiment 2, 200, 532, 1410, 3760 and 10 000 nM of JNJ-7706621 were combined with the same doses of ^{177}Lu-lilotomab satetraxetan as in experiment 1. Error bars: STDEV of triplicate samples.

Supplementary Figure 3

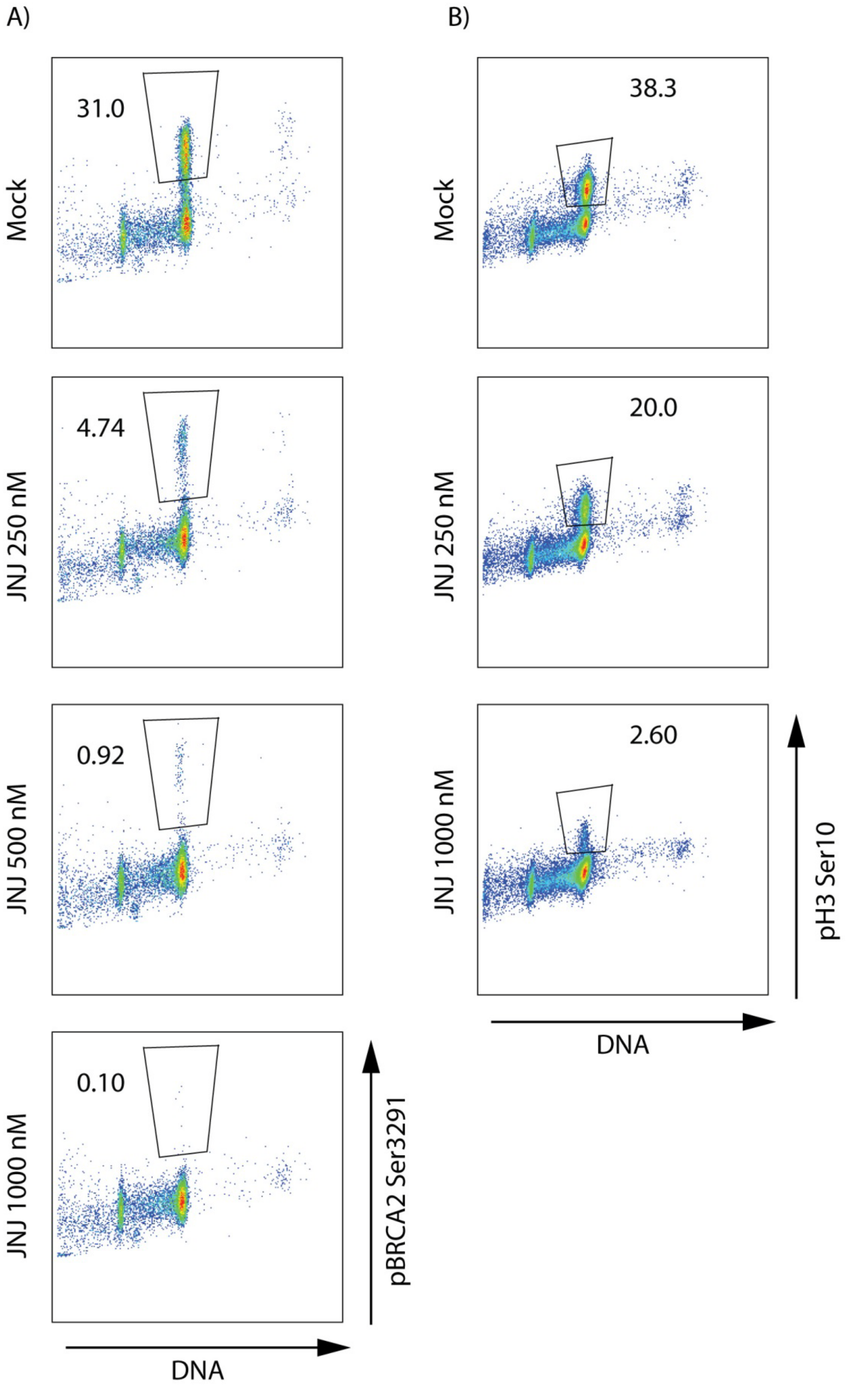

Supplementary Figure 3 JNJ-7706621 inhibits phosphorylation of CDK target BRCA2 and Aurora B target Histone 3 in mitosis

U-2932 cells were arrested in mitosis by treatment with 0.04 μg/ml nocodazol for 16 h. JNJ-7706621 was added to final concentrations of 250, 500 and 1000 nM. (A) Cells were harvested for flow analysis 6 h after adding inhibitor, and stained with anti-phospho-BRCA2Ser3291 and DNA-stain Hoechst. (B) Cells were harvested 1 h after adding inhibitor, and stained with anit-phospho-H3ser10 and DNA-stain FxCycleFar Red.

Supplementary Figure 4

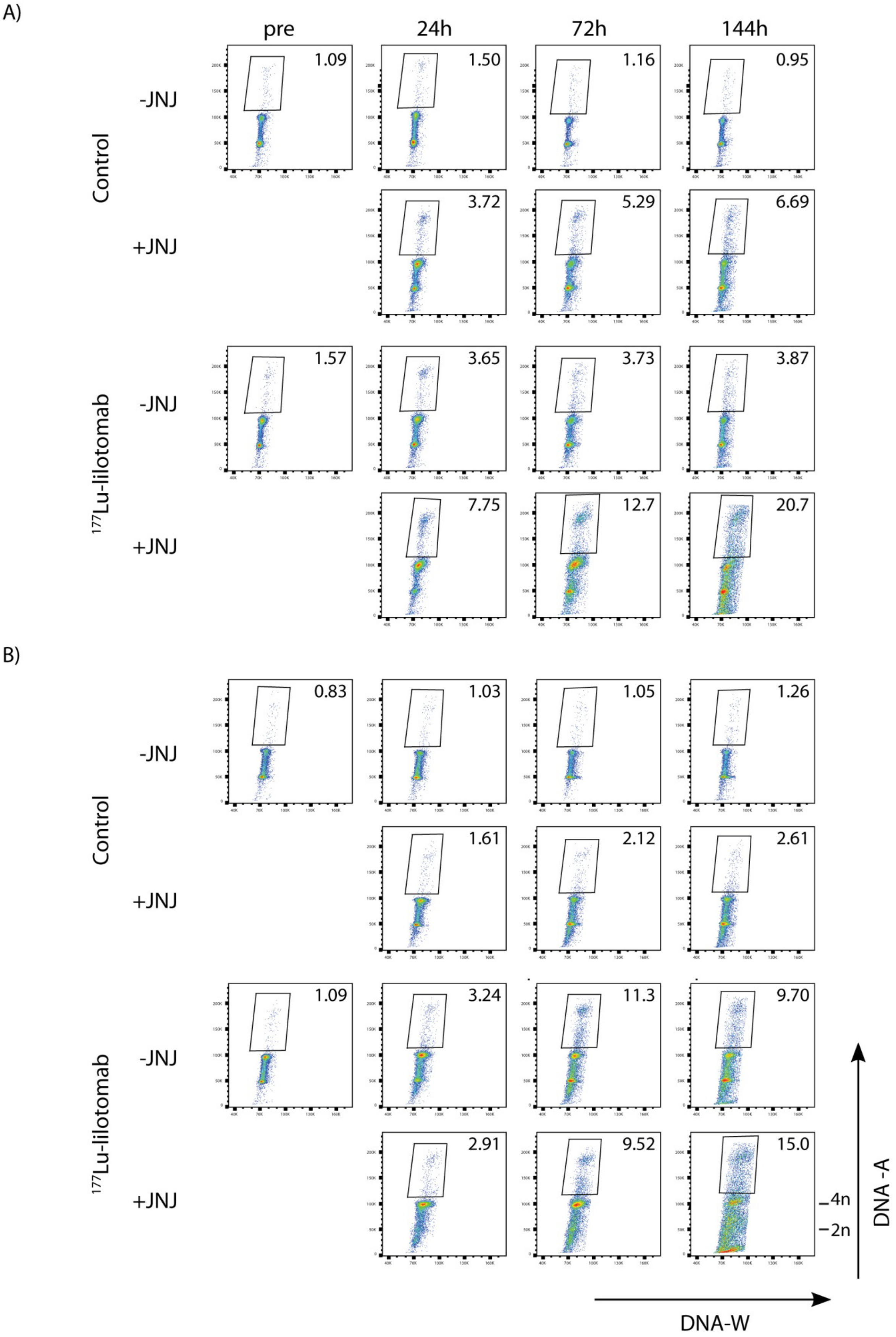

Supplementary Figure 4 JNJ-7706621 and ^{177}Lu-lilotomab satetraxetan in combination gives an increase in cells with DNA content above 4n

Data are from the same experiment as in Fig. 5. The width of FxCycleFar Red stained DNA (DNA-W) is plotted against the DNA area (DNA-A) revealing cell populations with DNA-A >4n. (A) U-2932 and (B) RIVA. Data shown are representative of two independent experiments.

Supplementary Figure 5

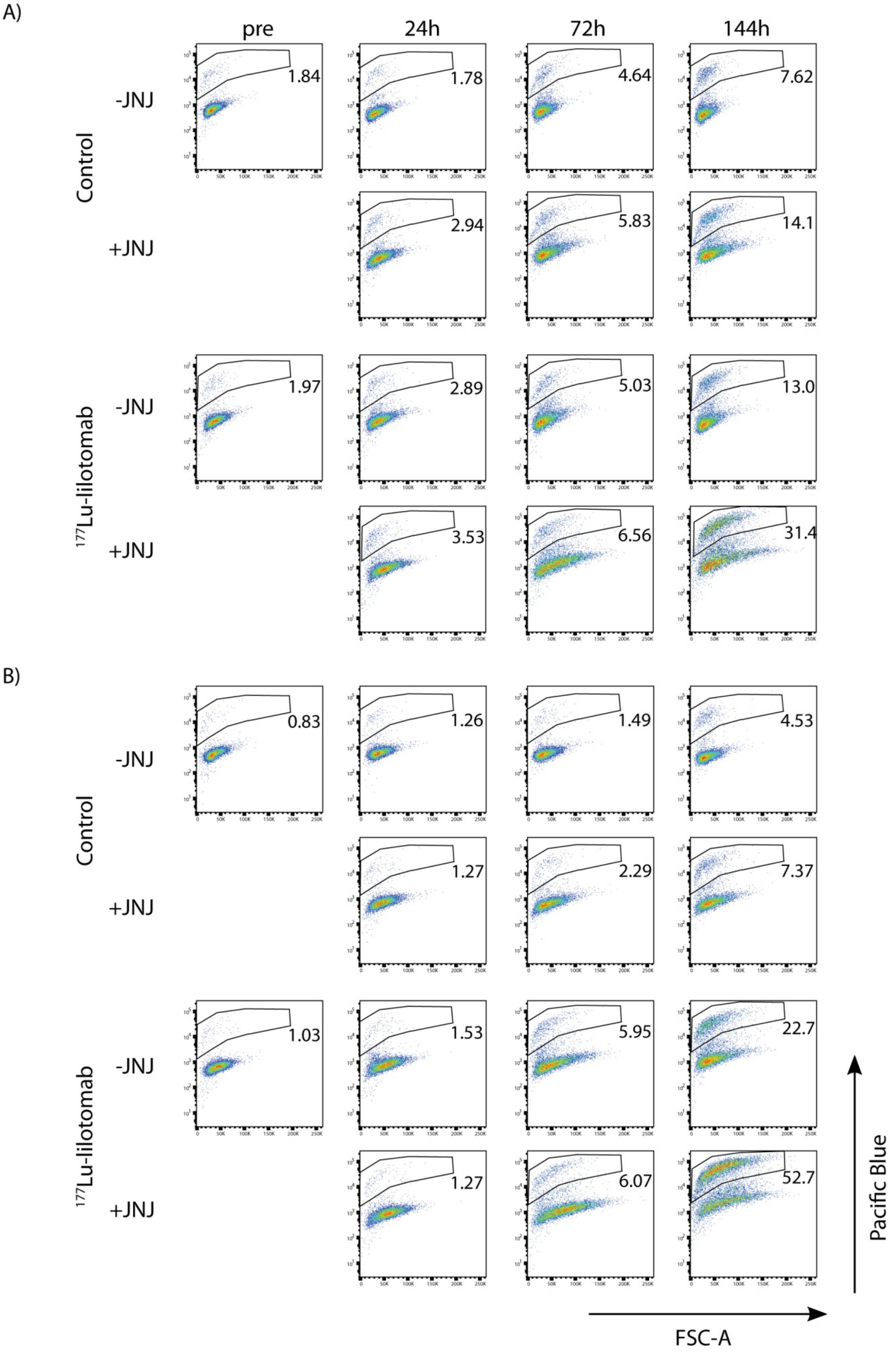

Supplementary Figure 5 JNJ-7706621 increases cell death and size of cells treated with ^{177}Lu-lilotomab satetraxetan

Data are from the same experiment as in Fig. 5. Forward scatter (FSC-A) has been plotted against Pacific Blue showing cell size and dead cells, respectively. (A) U-2932 and (B) RIVA. Data shown are representative of two independent experiments.

Supplementary Figure 6

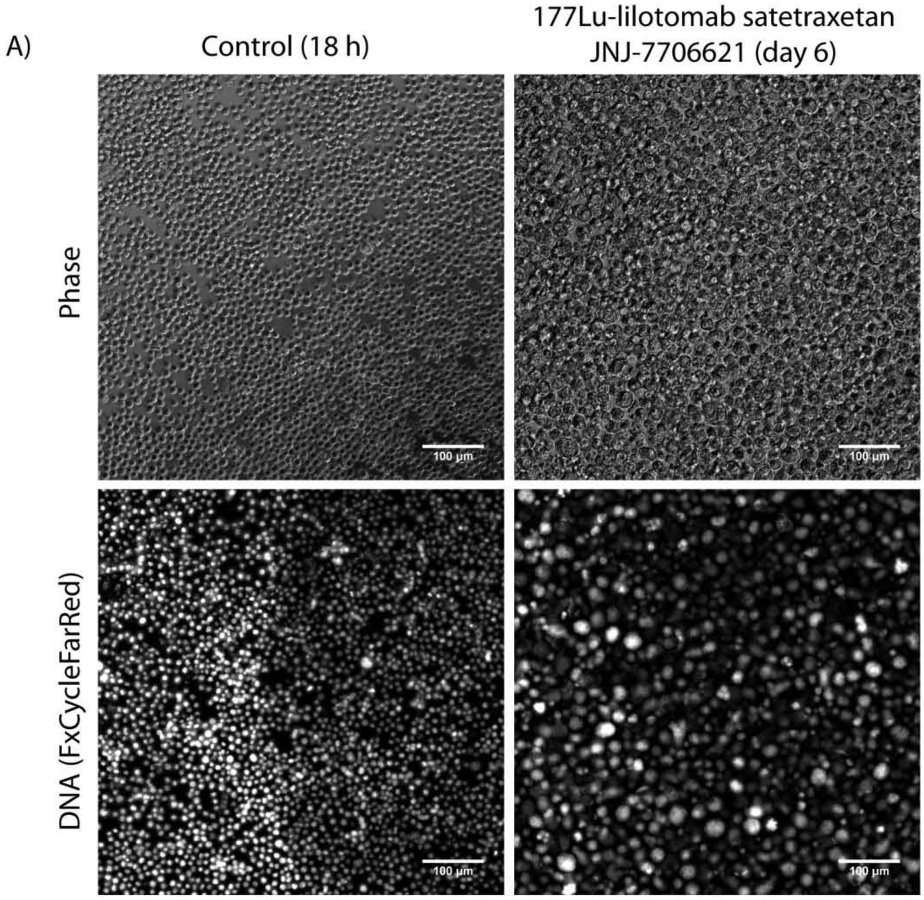

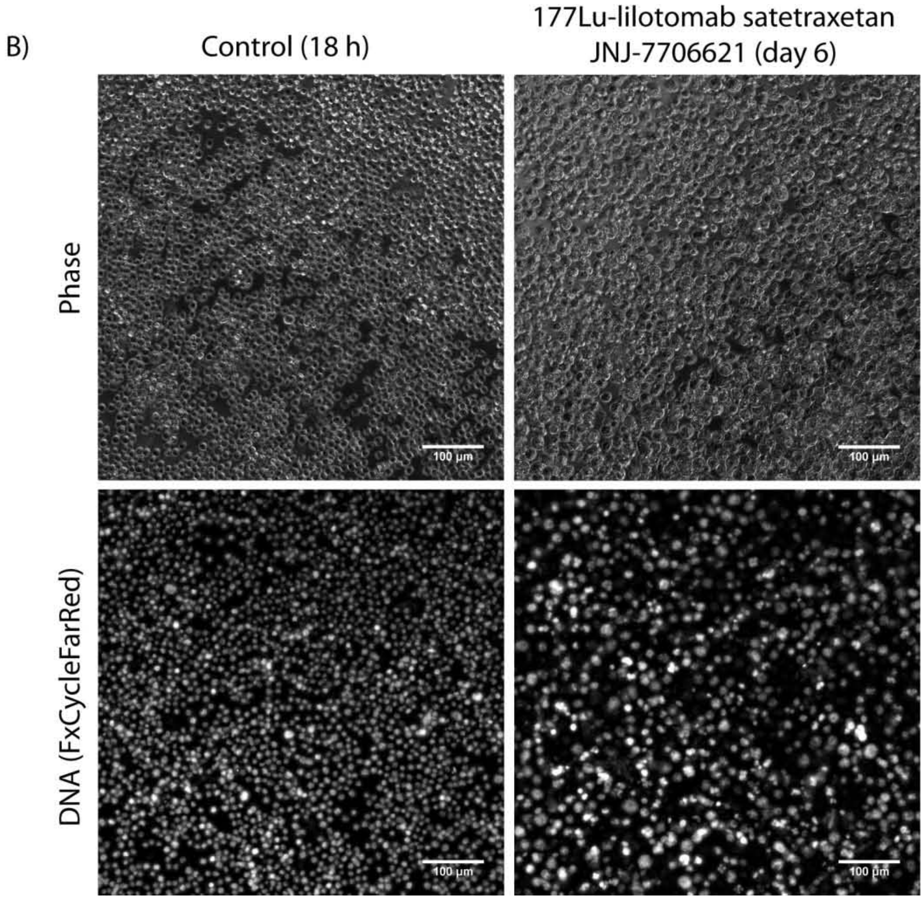

Supplementary Figure 6 Cells treated with a combination of JNJ-7706621 and ^{177}Lu-lilotomab satetraxetan appear multinucleated.

Microscopy pictures of cells from experiment in Fig. 5 showing multinucleation. (A) U-2932 (B) RIVA. Upper panels: Phase contrast. Lower panels: FxCycleFarRed stained DNA.

Supplementary Tables

Supplementary Table 1 Raw data from screen in U-2932

Excel spreadsheets with RLUs from the RealTime-Glo assay in the combinatorial drug screen in U-2932. Calculations of Bliss scores are also included. Worksheet 1: layout of drug plates indicating the location of each drug and controls. Worksheet 2: data from plate 1 at drug concentration 10 nM, Worksheet 3: data from plate 1 at drug concentration 1 µM. Worksheet 4: data from plate 2 at drug concentration 10 nM. Worksheet 5: data from plate 2 at drug concentration 1 µM.

Supplementary Table 2 Raw data from screen in RIVA

Excel spreadsheets with RLUs from the RealTime-Glo assay in the combinatorial drug screen in RIVA. The layout is the same as in Supplementary Table 1, except for the drug concentration in worksheet 3 and 5 being 100 nM.
